# Mucosal Immunization with Spore-Based Vaccines against *Mannheimia haemolytica* Enhances Antigen-Specific Immunity

**DOI:** 10.3390/vaccines12040375

**Published:** 2024-04-01

**Authors:** Muhammed Salah Uddin, Angelo Kaldis, Rima Menassa, José Ortiz Guluarte, Daniel R. Barreda, Le Luo Guan, Trevor W. Alexander

**Affiliations:** 1 Lethbridge Research and Development Centre, Agriculture and Agri-Food Canada, Lethbridge, AB T1J 4B1, Canada; salah.uddin@agr.gc.ca (M.S.U.); jose.ortizguluarte@agr.gc.ca (J.O.G.); 2 Department of Agricultural, Food and Nutritional Science, University of Alberta, Edmonton, AB T6G 2P5, Canada; dbarreda@ualberta.ca (D.R.B.); lguan@ualberta.ca (L.L.G.); 3 London Research and Development Centre, Agriculture and Agri-Food Canada, London, ON N5V 4T3, Canada; angelo.kaldis@agr.gc.ca (A.K.); rima.menassa@agr.gc.ca (R.M.); 4 Department of Biological Sciences, University of Alberta, Edmonton, AB T6G 2R3, Canada; 5 Faculty of Land and Food Systems, University of British Columbia, Vancouver, BC V6T 1Z4, Canada

**Keywords:** *Bacillus subtilis* spore, bovine respiratory disease, *Mannheimia haemolytica*, mucosal vaccine, spore-based vaccine

## Abstract

Background: *Mannheimia haemolytica* is a bovine respiratory pathogen commonly associated with bacterial bronchopneumonia. Current vaccine strategies have shown variable efficacy in feedlot cattle, and therefore novel vaccines are needed. *Bacillus subtilis* spores have been investigated as a mucosal vaccine platform, due to their ability to bind and present antigens to the mucosa and act as an adjuvant. The aim of this study was to develop two spore-based mucosal vaccines targeting *M. haemolytica* and evaluate their immunogenicity in mice. Methods: Two antigen constructs composed of cholera toxin B subunit, *M. haemolytica* leukotoxin, and either the *M. haemolytica* outer membrane protein PlpE (MhCP1) or GS60 (MhCP2) were synthesized, purified and then bound to spores as vaccines. In two separate mice trials, the spore-bound vaccines (Spore-MhCP1 and Spore-MhCP2) were administered to mice through intranasal and intragastric routes, while free antigens were administered intranasally and intramuscularly. Unbound spores were also evaluated intranasally. Antigen-specific serum IgG and mucosal IgA from bronchoalveolar lavage, feces, and saliva were measured after vaccination. Mice sera from all treatment groups were assessed for their bactericidal activity against *M. haemolytica*. Results: In both mice experiments, intramuscular immunization induced the strongest serum IgG antibody response. However, the intranasal administration of Spore-MhCP1 and Spore-MhCP2 elicited the greatest secretory IgA-specific response against leukotoxin, PlpE, and GS60 in bronchoalveolar lavage, saliva, and feces (*p* < 0.05). Compared to the intranasal administration of free antigen, spore-bound antigen groups showed greater bactericidal activity against *M. haemolytica* (*p* < 0.05). Conclusions: Since intranasally delivered Spore-MhCP1 and Spore-MhCP2 elicited both systemic and mucosal immune responses in mice, these vaccines may have potential to mitigate lung infection in cattle by restricting *M. haemolytica* colonization and proliferation in the respiratory tract. The efficacy of these mucosal spore-based vaccines merits further assessment against *M. haemolytica* in cattle.

## 1. Introduction

*Mannheimia haemolytica* is a key bacterial pathogen associated with bovine respiratory disease (BRD) [1,2,3]. In North America, the prevention and control of BRD in the beef cattle industry are managed primarily through vaccination and the mass use of antimicrobials at feedlot placement (metaphylaxis). However, current vaccines do not provide complete protection against infection [4,5], and the overuse of antimicrobials in livestock production has led to the emergence of antimicrobial resistance [3,6,7]. Although commercial vaccines for *M. haemolytica* are available, meta-analysis studies have revealed variable outcomes in field trials and demonstrated low efficacy in feedlots [5,8,9]. Thus, there is a need for the development of novel vaccines against *M. haemolytica* and other BRD pathogens, with increased efficacy.

In an attempt to enhance the effectiveness of vaccines, minimize the time needed to induce immunity, and lessen the stress involved with handling cattle, the development of mucosal BRD vaccines has been investigated [3,10]. Mucosal immunization can induce both mucosal and systemic immune responses, providing protection at the site of infection in addition to systemic immunity, and thus it can be advantageous over the parenteral delivery method [11]. A delivery system capable of preventing the loss of antigenicity is important for converting chimeric proteins into effective vaccines [12]. Over the last decade, numerous delivery systems, including microorganisms and nonliving nanoparticles, have been evaluated as adjuvants [13]. This includes probiotic bacteria which have a natural capacity to interact with the host mucosa and stimulate immune responses. Among them, the spore-forming Gram-positive bacterium *Bacillus subtilis* has been evaluated [13,14]. *B. subtilis* spores have several advantages for use as a vaccine vehicle including the ability to bind and present antigens to the mucosa as adjuvants [15,16] and high survivability in a variety of environmental conditions including the digestive tract. Studies have shown that mucosal immunization of mice with *B. subtilis* spores coated with antigens can induce strong antibody responses and provide protection against bacterial and viral infection [16,17,18,19]. The application of this spore-based technology to vaccinate cattle against BRD pathogens may have commercial benefits due to the low cost of inputs, well-established production systems, and the current use of *Bacillus* in livestock as feed additives. Thus, a low-cost, generally regarded as safe vaccine component with a proven record as an adjuvant would expedite industry adoption.

*M. haemolytica* virulence factors include outer membrane proteins, capsular polysaccharides, lipopolysaccharides, iron-binding proteins, and a ruminant-specific repeats in toxin—leukotoxin (LKT) [20]. The significance of these virulence factors in pathogenesis makes them the prime targets of the host immune response and, as a result, potential targets as vaccine components [7,21]. Multiple in vivo studies with LKT-deletion mutants have demonstrated that LKT is the primary virulence factor of *M. haemolytica* in pathogenesis [22,23,24] and, thus, LKT is considered one of the key elements for vaccine development [21]. Several studies have confirmed outer membrane lipoproteins as immunologically key surface antigens [25,26,27,28]. In the current study, two *M. haemolytica* chimeric proteins, MhCP1 and MhCP2, have been evaluated as components of candidate vaccines where LKT was retained as a core constituent. MhCP1 (CTB+PlpE+NLKT) is composed of truncated cholera toxin B subunit (CTB), the immune-dominant epitope (R2) region of the *M. haemolytica* outer membrane lipoprotein, PlpE, and the neutralizing epitope of LKT (NLKT) [29]. MhCP2 (CTB+LKT+GS60) comprises CTB, LKT, and another surface-exposed *M. haemolytica* outer membrane lipoprotein GS60. The purified chimeric proteins MhCP1 and MhCP2 were then adsorbed to *B. subtilis* spores, and the resultant spore-based vaccines termed “Spore-MhCP1” and “Spore-MhCP2” were evaluated in mice for the induction of immunogenicity. In a proof-of-concept study, we previously showed that *B. subtilis* spores coated with a dimer of *M. haemolytica* antigens, PlpE, and NLKT, can induce antibody responses after mucosal immunization in a mouse model [30]. The objectives of this current study were to develop two potential spore-based candidate vaccines, Spore-MhCP1 and Spore-MhCP2, against *M. haemolytica*, then assess the immunogenicity of these vaccines after delivery through mucosal (intranasal and intragastric) routes using mice models.

## 2. Materials and Methods

### 2.1. Spore Preparation

*B. subtilis* (RK28 strain) spores were used in this study and processed for antigen binding, as described previously [30]. Spores were enumerated by first suspending them in water and heating at 65 °C for 30 min to eliminate vegetative cells. The resulting suspension was then plated onto Luria–Bertani (LB) agar and incubated overnight, followed by the counting of colony-forming units, which indicated spore counts.

### 2.2. Chimeric Protein Purification

**Figure 1:**
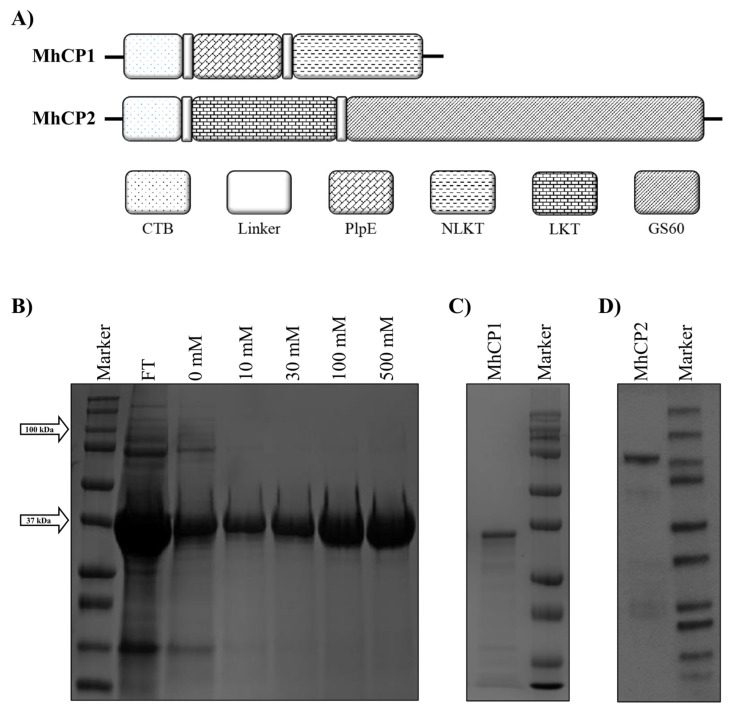
The construction and purification of chimeric proteins MhCP1 and MhCP2. (**A**) The modular architecture of MhCP1 and MhCP2. Both protein structures included truncated cholera toxin subunit B (CTB). MhCP1 contained epitopes of lipoprotein PlpE and neutralizing leukotoxin (NLKT); MhCP2 comprised a larger leukotoxin segment (LKT) and the surface-exposed *M. haemolytica* outer membrane lipoprotein GS60. (**B**) Eluted fractions of MhCP1, demonstrated by SDS-PAGE. (**C**) The purified recombinant chimeric protein MhCP1 demonstrated by Coomassie-stained SDS-PAGE. (**D**) The Coomassie-stained SDS-PAGE illustrated the purified chimeric protein MhCP2.

*M. haemolytica* chimeric proteins MhCP1 and MhCP2 were constructed as per the modular structure depicted in Figure 1A. MhCP1 comprised truncated cholera toxin B subunit (CTB), the immunodominant surface epitope (R2) region of the *M. haemolytica* outer membrane lipoprotein PlpE, and the neutralizing epitope of leukotoxin (NLKT). The chimeric protein MhCP1 had 305 amino acid (AA) residues, a calculated molecular weight of 34.01 kDa, and an isoelectric point (pI) of 9.0. Ayalew and colleagues previously used a comparable chimeric protein (CTB-R2-NLKT) similar to MhCP1, differing solely by the location of the His-tag, which was on the C-terminus end of their protein structure [29]. MhCP2 was constructed with CTB, LKT, and the surface-exposed outer membrane lipoprotein GS60. The LKT fragment used in MhCP2 consisted of the nucleotides encoding AA 705-953 of the *M. haemolytica* LktA gene (GenBank: M20730.1). The chimeric protein MhCP2 comprised 953 AA residues and had a calculated molecular weight of 104.83 kDa and a pI of 5.1. Sequences corresponding to the chimeric proteins, MhCP1 and MhCP2, were commercially synthesized (BioBasic Inc., Markham, ON, Canada), codon optimized for expression in *Escherichia coli*, and cloned into the pET28a vector. A polyhistidine tag (6 × His) was added at the N-terminal end of both sequences to facilitate purification and detection. The pET28a construct was transformed into *E. coli* BL21 Star (DE3), and the expression and purification of the recombinant chimeric protein MhCP1 was carried out according to previously described procedure [30]. The chimeric protein MhCP2 was purified under native conditions, without using any denaturant, as previously described [31]. After purification, the fractions containing the recombinant proteins were pooled and dialysed, and the buffer was exchanged with a storage buffer containing 20 mM Tris-HCl, pH 8.0. Subsequently, the proteins were concentrated by centrifugation using an Amicon Ultra centrifugal filter. The quality and identity of the purified proteins were assessed through sodium dodecyl sulfate–polyacrylamide gel electrophoresis (SDS-PAGE) and confirmed via Western blot analysis employing anti-His antibodies (ThermoFisher, Toronto, ON, Canada).

### 2.3. Chimeric Protein Adsorption to Spores

The conditions for optimizing spore adsorption with the chimeric proteins MhCP1 and MhCP2 were determined as described previously [17,30]. Briefly, varying amounts of antigen MhCP1 and MhCP2 (5 µg, 10 µg, and 50 µg), spores (5 × 10^8^ to 4 × 10^9^), buffer (citrate, phosphate buffered saline [PBS]), buffer pH (4 and 7), and temperature (4 °C, room temperature) were investigated to optimize antigen adsorption to spores. Following each set of conditions employed, the Spore-MhCP1 and Spore-MhCP2 mixtures were centrifuged and antigen binding to the spore coat, or remaining in supernatant, were evaluated by Western blotting using anti-His antibodies [30]. Conditions that resulted in the greatest amount of antigen adsorption to spore coats were considered optimal. For MhCP1, the following method was identified as optimal for spore adsorption and used in mice Experiment 1 (below): 2 × 10^9^ spores in water were centrifuged (5000× *g*; 5 min; 4 °C). The pellet was resuspended in PBS (pH 4) containing a total of 10 µg of purified MhCP1, and then mixed by inverting (1 h, 4°C). For antigen MhCP2 that was used in mice Experiment 2, the optimal binding conditions were similar, except 50 µg of MhCP2 was adsorbed to 2 × 10^9^ spores and citrate buffer (pH 4) was used.

### 2.4. Animals and Experimental Design

Animal experiments were carried out in strict accordance with the recommendations established in the Canadian Council on Animal Care Guidelines [32]. The experiments were reviewed and approved by the Lethbridge Research and Development Centre (LeRDC) Animal Care Committee (Animal Use Protocol # 1712) before commencement. Two separate studies were conducted to evaluate the immunogenicity of MhCP1 and MhCP2 in BALB/c mice (6 to 8 weeks old; Charles River Laboratories [Quebec, QC, Canada]). Prior to initiating the study, mice were acclimated to their cages and were housed in ambient conditions for temperature (22–25 °C) and a 12 h light/12 h dark cycle. Mice had free access to water and feed (Laboratory Rodent Diet 5001 [LabDiet, St. Louis, MO, USA]). A feeding and health log was maintained and the daily monitoring of animal health occurred throughout the experiments. In each experiment, mice were randomly assigned to the following six treatment groups (N = 12 per treatment): intramuscular free MhCP (IM:MhCP; positive control to measure the systemic immunity of antigens in free form); intranasal spore-bound MhCP (IN:Spore+MhCP); intranasal free MhCP without spore (IN:MhCP); intranasal spore only without any MhCP (IN:Spore); intragastric spore-bound MhCP (IG:Spore+MhCP); and control/naïve mice (Control; no MhCP administered, negative control). Details of the experimental design for both mice experiments are listed in Table 1.

### 2.5. Immunizations

Experiment 1: Mice were immunized through respective routes on day 0 and then received a booster immunization on day 21. Mice in the control treatment did not receive spores or antigen. Intramuscular immunization was performed on anaesthetized mice by mixing (1:1) purified antigen with Freund’s incomplete adjuvant (Sigma-Aldrich, Oakville, ON, Canada) and administering the mixture into thigh muscles. For intranasal groups, mice were lightly anaesthetized, and then respective vaccine formulations were administered in half doses by pipette tip to each nostril. The intragastric mice group was delivered the vaccine by oral gavage using a feeding tube (Instech Laboratories, Plymouth Meeting, PA, USA). The details of vaccine formulations, including the amount of recombinant protein MhCP1 and spore count (where applicable) per immunization time points, can be found in Table 1.

Experiment 2: Antigen MhCP2 was evaluated, where the inoculation procedures were identical to Experiment 1. For the treatment groups receiving MhCP2, either in free form (IM:MhCP2, IN:MhCP2) or in spore-bound antigens (IN:Spore+MhCP2, IG:Spore+MhCP2), a total of 50 µg of MhCP2 was administered at each vaccination time point as this was the optimal amount determined for spore adsorption.

### 2.6. Sample Collection

On days 21 and 42, blood, bronchoalveolar lavage (BAL), feces, and saliva were collected for both mice experiments. At each timepoint, six mice were sacrificed under deep anaesthesia. To collect freshly voided feces, each animal was placed into a separate cage for 3–5 min, or until feces was passed. Sterile oral swabs (Isohelix, Harrietsham, Kent, UK) were used for saliva collection thereafter. To obtain blood samples, anaesthesia was induced using isoflurane (4%) and maintained (2% isoflurane) using a SomnoSuite mask (Kent Scientific Corporation, Torrington, CT, USA). Blood was drawn from the caudal vena cava subsequent to opening the abdominal cavity. Mice were then euthanized humanly by exsanguination. The BAL samples were collected immediately after death by introducing a catheter into the trachea and instilling PBS into the bronchioles, followed by the recovery of BAL fluid. Antigen-specific IgG (blood) and IgA (feces, saliva, BAL) were quantified from samples to assess the immune response resulting from each vaccination route.

### 2.7. Antigen-Specific Serum IgG Quantification

Enzyme-linked immunosorbent assay (ELISA) was performed to quantify the antigen-specific antibody (IgG) responses in serum from both mice studies as previously described [30]. Sequences corresponding to individual components of the chimeric fusion constructs (i.e., PlpE, NLKT, LKT, and GS60) were synthesized commercially (BioBasic Inc., Markham, ON, Canada), codon optimized for expression in *E. coli*, and then purified by immobilized metal affinity chromatography. Purified recombinant antigen components PlpE, NLKT, LKT, and GS60 were utilized as ligands to coat ELISA plates individually at a concentration of 50 ng/well, and the plates were incubated overnight at 4 °C. After a wash step the next morning and the addition of Blocking Solution (50 mM Tris, 0.14 M NaCl, 1% BSA, pH 8.0), the plates were incubated (1 h, room temperature). Serum samples were diluted (50 mM Tris, 0.14 M NaCl, 1% BSA, 0.05% Tween 20), and aliquots (100 µL) of each dilution were pipetted into wells in duplicate, followed by incubation (1 h, room temperature). Horseradish peroxidase-conjugated anti-mouse IgG (Cat. No. A90-131P; Bethyl Laboratories Inc., Montgomery, TX, USA) was used as the secondary antibody. After introducing the secondary antibody, plates were incubated at room temperature for 1 h, followed by colour development using TMB (3,3′,5,5’-tetramentylbenzidine; ThermoFisher, Toronto, ON, Canada). Reactions were stopped by ELISA stop solution (0.2 M H_2_SO_4_), and optical densities of the reactions were measured at a wavelength of 450 nm on a Synergy HTX multi-detection microplate reader (BioTek Instruments Inc., Winooski, VT, USA) with Gen5 analysis software version 3.05 (BioTek Instruments Inc., Winooski, VT, USA). Concentrations were determined using a standard curve established with a Mouse IgG ELISA Quantitation Kit (Cat. No. E90-131; Bethyl Laboratories Inc., Montgomery, TX, USA), utilizing Mouse Reference Serum (RS10-101-6; Bethyl Laboratories Inc.) as the standard.

### 2.8. Antigen-Specific Secretory IgA (sIgA) Quantification

BAL, feces, and oral swab samples were pre-processed for IgA quantification, as described previously [33]. After pre-processing, ELISA was used to measure the concentrations of mucosal secretory IgA (sIgA) as previously described [30]. Briefly, 96-well ELISA plates were coated with 50 ng/well of purified recombinant antigen components NLKT, PlpE, LKT, and GS60 independently and incubated at 4 °C overnight. Incubation in Blocking Solution was conducted, as described for IgG, and then duplicate samples were pipetted into individual plate wells, followed by incubation at room temperature (1 h). Plates were washed, and the HRP conjugated anti-Mouse IgA Detection Antibody (A90-103P; Bethyl Laboratories Inc., Montgomery, TX, USA) was added, followed by an additional incubation period at room temperature (1 h). TMB substrate was added, and after colour development, the reaction was stopped by the addition of stop solution. The plates were read at 450 nm to determine optical density on a Synergy HTX multi-detection microplate reader (BioTek Instruments Inc., Winooski, VT, USA).

### 2.9. Complement-Mediated Serum Bactericidal Activity (SBA)

Sera collected on day 42 were evaluated for SBA against *M. haemolytica*, as described previously [34]. The SBA assay utilized neonatal colostrum-deprived calf serum (CDCS) as an external complement source, which was previously determined to have limited bactericidal activity against *M. haemolytica*. The CDCS and mice sera were tested in triplicate for bactericidal activity in the presence of complement. Endogenous complement activity of sera was eliminated by heat inactivation (56 °C, 30 min) prior to analysis. A serotype 1 strain of *M. haemolytica* was used for the assay. To enhance surface epitope exposure by eliminating the polysaccharide capsule, *M. haemolytica* cells underwent decapsulation through incubation at 41 °C with gentle shaking at 100 rpm for 1 h. The decapsulated *M. haemolytica* were suspended in PBS to an optical density of 0.50 at 600 nm and a 1:1000 dilution was then used for SBA. For this, mice sera (25 µL; heat-inactivated), exogenous complement (25 µL), and decapsulated *M. haemolytica* (25 µL) were combined and plated in triplicate, followed by incubation for 30 min (37 °C). Suspensions from the beginning of the experiment (T0) and after 30 min of incubation (T30) were plated and cultured overnight (37 °C with 5% CO_2_), followed by bacterial enumeration. Bactericidal activity was determined as the percentage of cells killed and calculated as follows: [(T0 growth − T30 growth)/T0 growth] × 100%. Negative controls in each SBA assay consisted of the complement source alone, without antibodies (mice serum).

### 2.10. Statistical Analysis

The analysis of ELISA and SBA assay was conducted using one-way ANOVA. Statistical significance was assessed against the control through one-way ANOVA with Tukey’s post-test. Statistical significance was considered when a *p*-value was less than 0.05. Microsoft Excel 2010 and GraphPad Prism version 10.1.0 were utilized for all analyses.

## 3. Results

### 3.1. Adsorption of Chimeric Proteins to Spores

The modular structures of MhCP1 and MhCP2 are shown in Figure 1A. Coomassie-stained SDS-PAGE demonstrated the size, purity, and integrity of the recombinant protein MhCP1 (Figure 1B,C) and MhCP2 (Figure 1D). For Spore-MhCP1, the best spore-binding condition was achieved when 10 µg of antigen MhCP1 was mixed with 2 × 10^9^ spores in PBS at pH 4, as indicated by the presence and absence of a band corresponding to MhCP1 from the spore coat and supernatant, respectively, in Western blots (Supplementary Figure S1A). Spore-MhCP2 displayed the best binding condition when 50 µg of antigen MhCP2 was mixed with 2 × 10^9^ spores in citrate buffer at pH 4, as indicated by the band from Western blots (Supplementary Figure S1B). Among the tested parameters, both MhCP1 and MhCP2 were efficiently adsorbed onto the spore coat at pH 4 and the binding remained most stable at 4 °C compared to room temperature, whereas at pH 7, low levels of binding were observed. Freshly prepared Spore-MhCP1 and Spore-MhCP2 (within one hour of their preparation) using optimized binding conditions were used for the immunization of mice.

### 3.2. Experiment 1. Antigen-Specific Antibody Production from Spore-MhCP1

**Figure 2:**
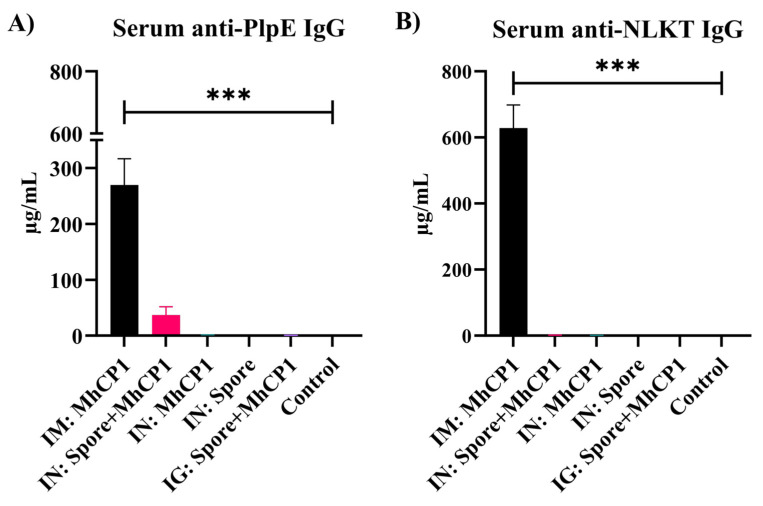
Experiment 1—Antigen-specific IgG concentrations in mice sera on day 42. Mice were vaccinated on day 0 and then received a booster immunization on day 21. The treatment groups were: intramuscular (IM:MhCP1), intranasal spore-bound antigen (IN:Spore+MhCP1), intranasal antigen only (IN:MhCP1), intranasal spore only (IN:Spore), oral/intragastric spore-bound antigen (IG:Spore+MhCP1), and control/naïve mice (Control). IgG specific to PlpE (**A**) and NLKT (**B**) are shown (mean ± SEM). *** *p* < 0.001.

**Figure 3:**
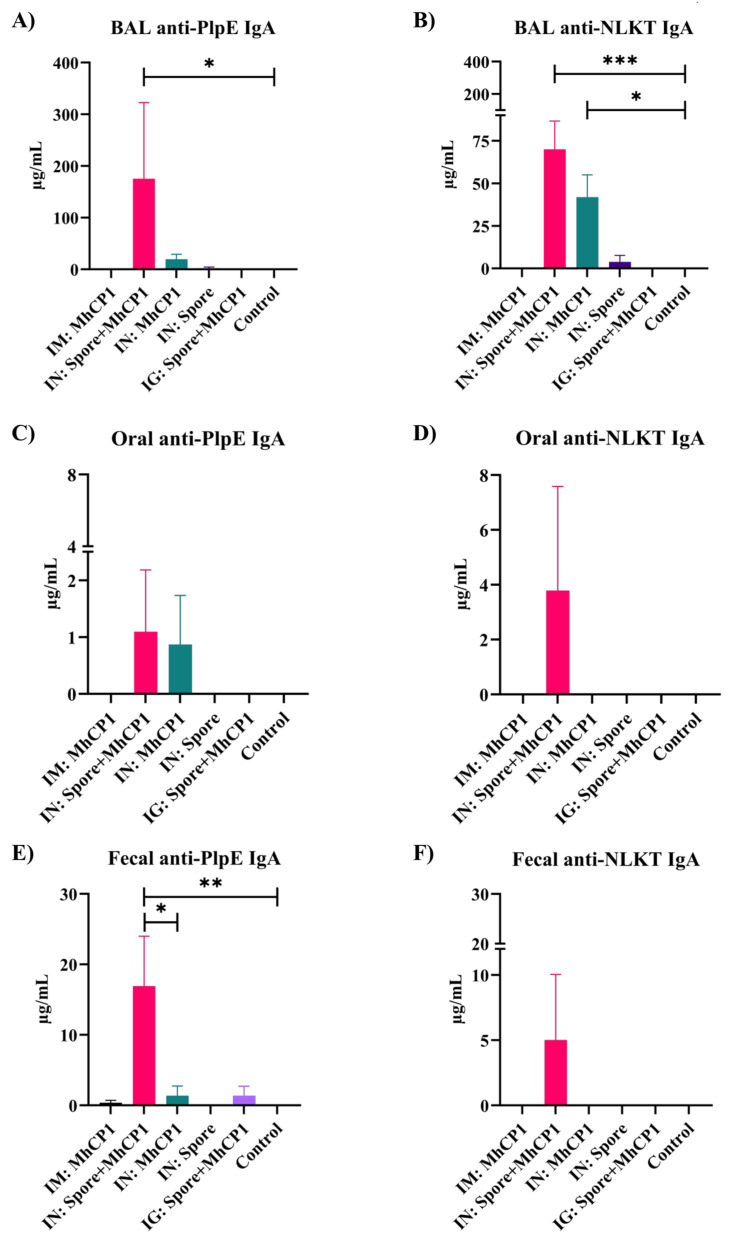
Experiment 1—Enhanced mucosal immunity induced by Spore-MhCP1 in mice on day 42. Anti-PlpE IgA and anti-NLKT IgA antibody concentrations in mucosa samples from mice on day 42 are shown for the following treatment groups: intramuscular (IM:MhCP1), intranasal spore-bound antigen (IN:Spore+MhCP1), intranasal antigen only (IN:MhCP1), intranasal spore only (IN:Spore), oral/intragastric spore-bound antigen (IG:Spore+MhCP1), and control/naïve mice (Control). Mice were vaccinated on day 0 and then received a booster immunization on day 21. IgA was measured in bronchoalveolar lavage (BAL), feces, and oral swab samples (mean ± SEM). * *p* < 0.05, ** *p* < 0.01, and *** *p* < 0.001.

The vaccination of mice with free MhCP1 and Spore-MhCP1 induced anti-PlpE and anti-NLKT antibodies (Figure 2 and Figure 3). This indicated that MhCP1 was immunogenic and retained immunogenicity after binding to spores. On day 21, after receiving a single immunization, IM:MhCP1 treatment mice had a greater serum IgG immune response for PlpE (*p* < 0.05; Supplementary Figure S2A) and NLKT (*p* < 0.05; Supplementary Figure S2B), when compared to other treatments. On day 42, subsequent to the booster vaccination, concentrations of IgG increased several fold in IM:MhCP1 mice (Figure 2). Apart from IM:MhCP1 mice, the spore-bound intranasal group (IN:Spore+MhCP1) also followed a similar trend and showed numerically greater serum anti-PlpE antibody concentrations, as compared to other mice treatments on day 21 (*p* > 0.05; Supplementary Figure S2A) and day 42 (*p* > 0.05; Figure 2A). No other treatment group showed serum antibody production on days 21 or 42 above the ELISA limit of detection (≥7.8 ng/mL).

On day 21, with a single dose of immunization, all the intranasal treatment groups showed secretory IgA immune responses in BAL samples against PlpE (Supplementary Figure S2C) and NLKT (Supplementary Figure S2D). However, on day 21, IgA immune responses were not observed for feces and oral swab samples (limit of detection ≥ 7.8 ng/mL) from any treatment groups. On day 42, BAL samples from IN:Spore+MhCP1 mice had greater secretory anti-PlpE and anti-NLKT IgA concentrations compared to other treatment groups (*p* < 0.05; Figure 3A,B). IN:MhCP1 treatment mice exhibited numerically higher anti-PlpE (*p* > 0.05; Figure 3A) and anti-NLKT IgA (*p* < 0.05; Figure 3B) in BAL samples while compared to control mice. Interestingly, one out of six mice from the IN:Spore only treatment group showed minimal levels of anti-PlpE and anti-NLKT IgA in BAL samples. None of the mice from the IM:MhCP1, IG:Spore+MhCP1, and control treatment groups had a detectable secretory antibody in BAL fluid. Compared to the control treatment group, IN:Spore+MhCP1 treatment mice had increased levels of anti-PlpE IgA in fecal samples (*p* < 0.05; Figure 3E) and numerically higher levels of anti-PlpE IgA in oral swabs (*p* > 0.05; Figure 3C). The IM:MhCP1, IN:MhCP1, and intragastric Spore+MhCP1 group showed minimal levels of anti-PlpE IgA in feces (Figure 3E). The IN:Spore+MhCP1 treatment mice group was the only group that showed anti-PlpE and anti-NLKT IgA in all the mucosal samples evaluated. Despite NLKT and PlpE being delivered to mice as a chimera protein, immune responses to each antigen component were differential.

### 3.3. Experiment 2. Antigen-Specific Antibody Production from Spore-MhCP2

**Figure 4:**
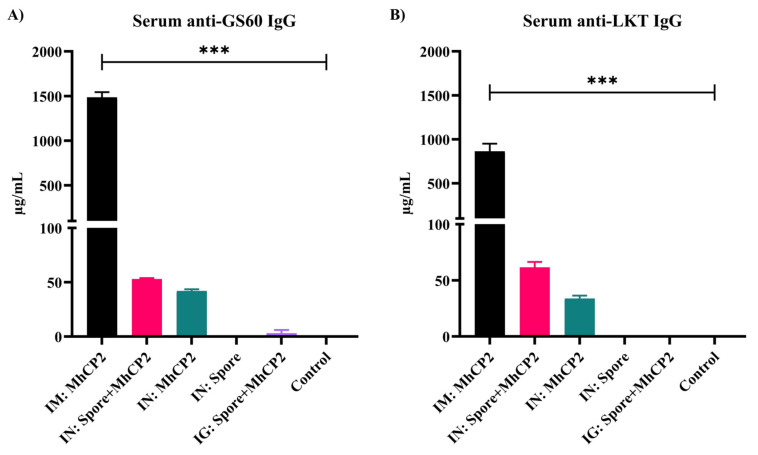
Experiment 2—Antigen-specific IgG concentrations in mice sera on day 42. Mice were vaccinated on day 0 and then received a booster immunization on day 21. The treatment groups were: intramuscular (IM:MhCP2), intranasal spore-bound antigen (IN:Spore+MhCP2), intranasal antigen only (IN:MhCP2), intranasal spore only (IN:Spore), oral/intragastric spore-bound antigen (IG:Spore+MhCP2), and control/naïve mice (Control). IgG specific to GS60 (**A**) and LKT (**B**) are shown (mean ± SEM). *** *p* < 0.001.

**Figure 5:**
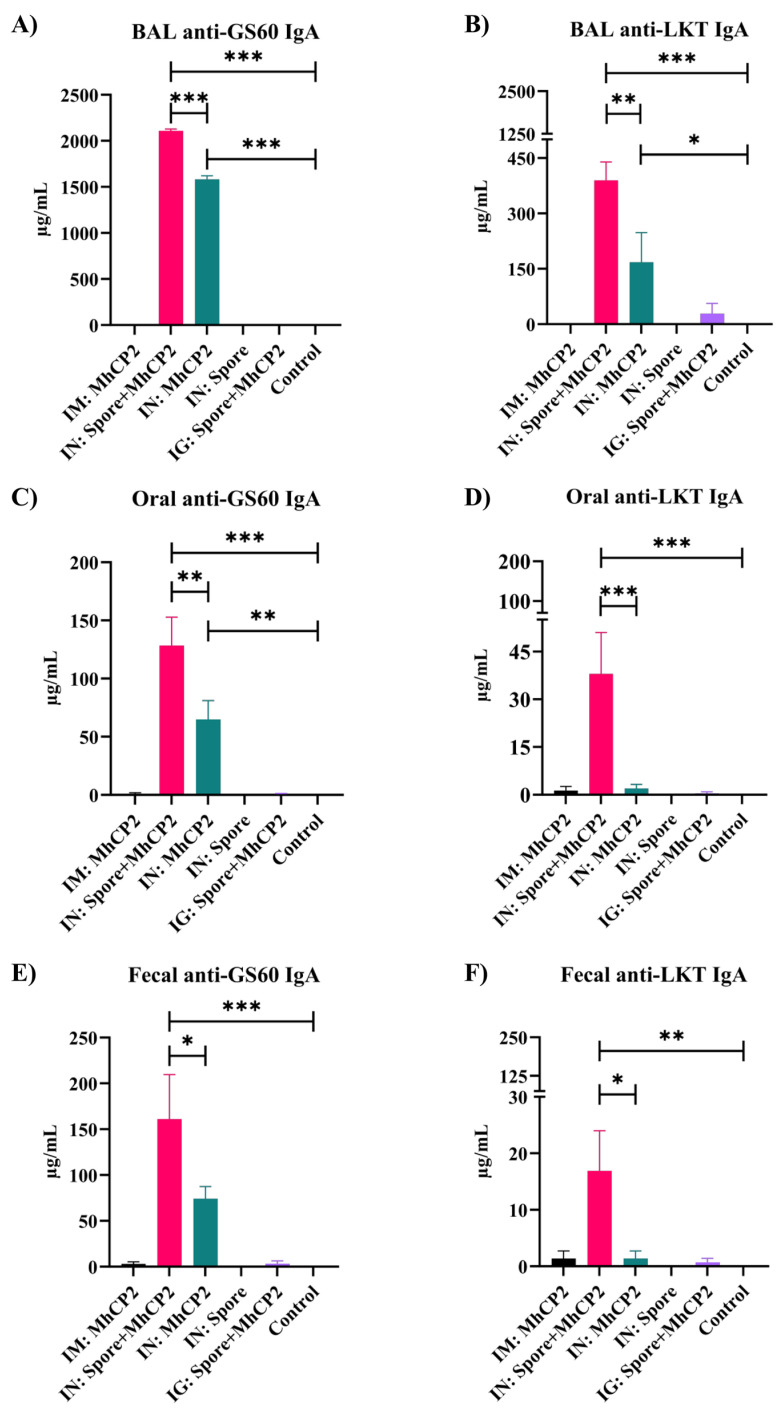
Experiment 2—Enhanced mucosal immunity induced by Spore-MhCP2 in mice on day 42. Anti-GS60 IgA and anti-LKT IgA antibody concentrations in mucosa samples from mice on day 42 are shown for the following treatment groups: intramuscular (IM:MhCP2), intranasal spore-bound antigen (IN:Spore+MhCP2), intranasal antigen only (IN:MhCP2), intranasal spore only (IN:Spore), oral/intragastric spore-bound antigen (IG:Spore+MhCP2), and control/naïve mice (Control). Mice were vaccinated on day 0 and then received a booster immunization on day 21. IgA was measured in bronchoalveolar lavage (BAL), feces, and oral swab samples (mean ± SEM). * *p* < 0.05, ** *p* < 0.01, and *** *p* < 0.001.

Vaccinating mice with both unbound MhCP2 and Spore-MhCP2 resulted in the generation of antibodies against GS60 and LKT, demonstrating that the chimeric antigen MhCP2 retained its immunogenicity following adsorption onto spores (Figure 4 and Figure 5).

After receiving a single immunization, on day 21, IM:MhCP2 treatment mice exhibited a greater serum IgG immune response as compared to other treatment mice against GS60 (*p* < 0.05; Supplementary Figure S3A) and LKT (*p* < 0.05; Supplementary Figure S3B). Although limited in titres, IN:Spore+MhCP2 treatment mice also stimulated serum anti-GS60 and anti-LKT IgG with a single dose of vaccination (Supplementary Figure S3A,B). After receiving the booster dose, the IgG immune responses increased several fold both in IM:MhCP2 and IN:Spore+MhCP2 treatment mice by day 42 and remained higher compared to other treatment mice (*p* < 0.05; Figure 4). IN:MhCP2 treatment mice also exhibited serum anti-GS60 and anti-LKT IgG, although to a lesser extent while compared to IN:Spore+MhCP2 treatment mice on day 42 (*p* > 0.05; Figure 4). IN:Spore and control treatment mice did not show any detectable serum antibody production (detection limit ≥ 7.8 ng/mL) on day 42.

The IN:Spore+MhCP2 treatment group showed a greater secretory IgA immune response compared to other treatments in BAL samples against GS60 (*p* < 0.05; Supplementary Figure S3C) and LKT (*p* < 0.05; Supplementary Figure S3D) on day 21, after a single dose of immunization. Compared to control mice, the IN:Spore+MhCP2 treatment group also exhibited a greater anti-GS60 IgA in saliva and feces (*p* < 0.05; Supplementary Figure S3E,G). Although the IN:MhCP2 group also stimulated anti-GS60 IgA in BAL, saliva, and feces on day 21, this was to a lesser extent than the IN:Spore+MhCP2 treatment mice (Supplementary Figure S3C,E,G). On day 42, after receiving the booster dose, immune responses increased several fold, compared to day 21, both in IN:Spore+MhCP2 and IN:MhCP2 treatment mice (Figure 5). The IN:Spore+MhCP2 resulted in greater secretory anti-GS60 and anti-LKT IgA compared to other treatment groups in all the mucosal samples evaluated (*p* < 0.05; Figure 5A–F). Apart from IN:Spore+MhCP2 treatment mice, IN:MhCP2 also exhibited a production of anti-GS60 IgA in BAL and saliva (*p* < 0.05; Figure 5A,C) and anti-LKT IgA in BAL samples while compared to control treatment mice (*p* < 0.05; Figure 5B). IN:Spore and control treatment mice did not show any detectable IgA antibody levels in any of the samples evaluated (detection limit ≥ 7.8 ng/mL) at any time points. IN:Spore+MhCP2 and IN:MhCP2 treatment groups showed anti-GS60 and anti-LKT IgA in all the mucosal samples evaluated, although the IN:Spore+MhCP2 treatment group consistently had greater mucosal titres compared to the IN:MhCP2 treatment group for each sample evaluated (*p* < 0.05; Figure 5). This finding demonstrated that despite the fact that GS60 and LKT were administered as a chimera, each antigen constructs elicited distinct immune responses and the spore enhanced immunogenicity.

### 3.4. Complement-Mediated SBA

**Figure 6:**
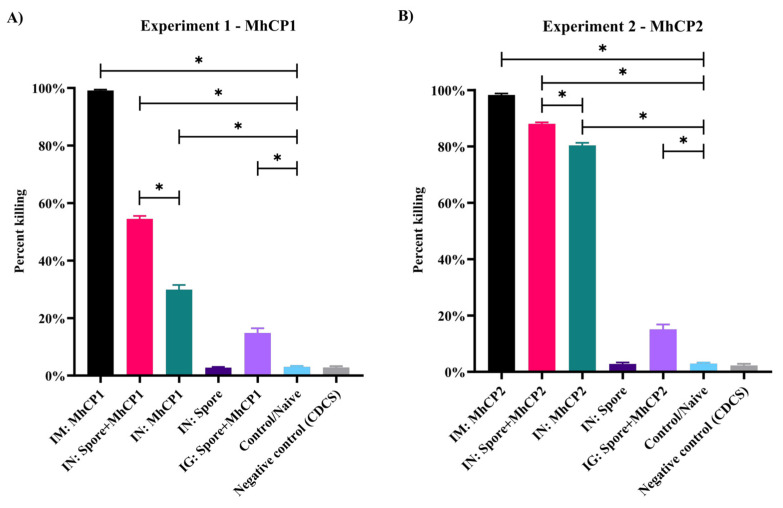
Serum bactericidal activity (SBA) assay, from samples collected on day 42. Antibodies present in the mice sera from Experiment 1 (**A**) and Experiment 2 (**B**) were assessed for their bactericidal activity against *M. haemolytica* through the complement-dependent SBA assay. Bactericidal activity from both experiments are shown for the following treatment groups: intramuscular (IM:MhCP), intranasal spore-bound antigen (IN:Spore+MhCP), intranasal antigen only (IN:MhCP), intranasal spore only (IN:Spore), oral/intragastric spore-bound antigen (IG:Spore+MhCP), and control/naïve mice (Control). The negative control in each SBA assay involved using only the complement source without the addition of antibodies (mice serum). * *p* < 0.05.

Control and IN:Spore treatment mice in both experiments, and CDCS used as complement, had minimal bactericidal activity (Figure 6). In Experiment 1, IM:MhCP1 mice sera killed more *M. haemolytica* (99.1%), compared to other treatments (*p* < 0.05; Figure 6A). Both IN:Spore+MhCP1 and IN:MhCP1 treatment mice showed greater bactericidal activity (54.5% and 29.9%, respectively) than control mice (*p* < 0.05; Figure 6A). Interestingly, the IG:Spore+MhCP1 treatment group also showed a level (14.8%) of bactericidal activity. In Experiment 2, sera from IM:MhCP2 treatment mice displayed higher bactericidal activity (99.1%), followed by IN:Spore+MhCP2 (88.0%) and IN:MhCP2 (80.1%) treatment mice in comparison to control mice (*p* < 0.05; Figure 6B). The IG:Spore+MhCP2 treatment group also showed limited but significant bactericidal activity (15.1%) versus sera from control mice (*p* < 0.05; Figure 6B). Compared to the intranasal administration of free antigen, the intranasal spore-bound antigen group showed greater bactericidal activity against *M. haemolytica* (*p* < 0.05) in both experiments.

## 4. Discussion

In this study, two spore-based vaccine candidates were developed, using *M. haemolytica* chimeric proteins MhCP1 (CTB+PlpE+NLKT) and MhCP2 (CTB+LKT+GS60). Both vaccines were able to stimulate antigen-specific immune responses after the immunization of mice. In support of this, we previously showed that a larger dimer repeat of NLKT and PlpE (56.4 kDa, pI of 9.1) could bind to spores and remain immunogenic, similar to MhCP1 [30]. The versatility of chimeric proteins in delivering multiple antigens to the immune system is advantageous in vaccine development [35]. The chimeric protein MhCP1 encoded the neutralizing epitope of LKT, which is the principal virulence factor of *M. haemolytica* and an immunodominant epitope region (R2) of the outer membrane lipoprotein, PlpE [29]. A truncated CTB (lacking signal peptide), which can also be found in approved human mucosal vaccines [36], was fused to the N-terminus as a mucosal adjuvant. When administered through intranasal or rectal routes, it has been demonstrated in human studies that CTB effectively triggers the induction of antigen-specific local IgA and systemic IgG responses [36,37]. Similarly, MhCP2 consisted of the truncated CTB fused to LKT and a surface-exposed *M. haemolytica* outer membrane lipoprotein, GS60. Both of these outer membrane proteins, PlpE and GS60, are reported to be strongly immunogenic [21] and conserved across *M. haemolytica* serotypes including serotypes 1 and 6 which are most often associated with BRD morbidity and mortality [7,21]. Keeping CTB and LKT as core components, two established immunogenic constructs (PlpE and GS60) were chosen to formulate the chimeric proteins. This resulted in two chimeric proteins that differed both in size and amino acid composition.

In order to establish the most effective adsorption conditions, a comparative analysis of spore and chimeric protein binding was conducted with various quantities of each component, under varied buffer pH levels and reaction temperatures. Previous research has demonstrated that proteins exhibit enhanced binding for negatively charged bacterial spores when the pH of the aqueous environment drops below the isoelectric point (pI) of the proteins [17,19]. Given that the pI values for MhCP1 and MhCP2 were 9.0 and 5.1, respectively, binding experiments were conducted at both pH 4 and pH 7. MhCP1 and MhCP2 exhibited optimal adsorption onto the spore coat at pH 4, while reduced binding levels were observed at pH 7. This observation supports findings from our earlier study involving a dimer of a PlpE-NLKT construct [30] and is consistent with other studies [16,17] suggesting that the extent of protein adsorption to spores is reliant upon both solution pH and protein structure. While the complete molecular mechanisms underlying spore adsorption remain elusive, the current understanding suggests that the isoelectric point, electric charge, and the relative hydrophobicity of the antigen play pivotal roles in facilitating efficient adsorption [17,38]. Despite the efficient adsorption of both MhCP1 and MhCP2 onto the spore coat at pH 4, notable distinctions were observed in the reaction buffers used. Specifically, MhCP1 demonstrated the highest adsorption efficacy when employing PBS buffer (pH 4), whereas MhCP2 exhibited optimal binding using citrate buffer (pH 4) in the binding experiment. The differences between the protein structures and their pI values necessitated two different buffers to be used to establish the optimal adsorption conditions. It was also necessary to use a higher amount of MhCP2 (five times compared to MhCP1), to achieve optimal adsorption.

Previously, Ayalew et al. (2009) conducted a study with the same chimeric protein as MhCP1 and revealed that the components were immunogenic [29]. This supports our study, where the generation of immune responses against NLKT and PlpE were observed in mice, following intramuscular immunization with free MhCP1. Despite MhCP1 being developed as a chimeric protein, NLKT and PlpE components yielded seroconversion independently. When we tested three different routes either with free or spore-bound MhCP1 in mice, our results demonstrated that the MhCP1 antigen adsorbed to spores in a manner that preserved its immunogenicity. While the IM:MhCP1 treatment group was used as a positive control and showed strong seroconversion, the main goal of this study was to develop Spore-MhCP1 as a mucosal vaccine candidate and to investigate if this spore-bound vaccine could enhance the immune response compared to the free form of this antigen. Mice immunization via the intranasal route with Spore-MhCP1 induced mucosal immunity, as evidenced by the production of secretory IgA against *M. haemolytica* NLKT and PlpE. Although IN:Spore+MhCP1 did not produce an IgG response as strong as that induced by intramuscularly administered free MhCP1, it was the only treatment group other than IM:MhCP1 to elicit an IgG response. In addition, IN:Spore+MhCP1 was the sole treatment group to result in specific IgA responses against both PlpE and NLKT antigens at all mucosal sites evaluated and with significantly greater IgA levels in BAL and fecal samples. Thus, IN:Spore+MhCP1 immunization resulted in both mucosal and systemic immune responses. Intranasal immunization with free MhCP1 (IN:MhCP1) also stimulated anti-PlpE IgA in all the mucosal samples evaluated, but at a lesser extent than IN:Spore+MhCP1. This indicated that binding MhCP1 to *Bacillus* spores did result in an increased immune response and highlighted that the spores were effective as adjuvant. Mucosal immunity is shared between different mucosae [10], and our study showed that intranasal immunization can result in secretory antibodies in both the oral cavity and gut.

Batra and colleagues formulated a BHV-1-vectored vaccine expressing PlpE-LKT chimeric proteins and showed the generation of antibodies in bighorn sheep following intranasal vaccination [39]. However, antibody production against surface antigen was inconsistent, and the vaccine was not effective in protecting against *M. haemolytica* challenge. An intranasal vaccination of cattle with the same MhCP1 chimeric protein used in our study stimulated serum and nasal antibodies in vaccinated calves, and intrabronchial challenge in these calves displayed fewer clinical symptoms than unvaccinated animals [29]. Given the enhanced immune responses from IN:Spore+MhCP1 compared to IN:MhCP1 in our study, we hypothesise that the intranasal immunization of this Spore-MhCP1 vaccine may confer increased protection against *M. haemolytica* infection in cattle. The intragastric administration of spore-bound MhCP1 was able to generate a limited IgA immune response against PlpE in fecal samples, and this finding is consistent with a previous report where an immune response was reported in fecal samples after oral immunization with spore-bound antigen [30]. Dilution of antigens in the stomach and degradation due to the low gastric pH and the presence of proteases in the gastrointestinal tract are possible contributing factors that reduce the antigenicity of intragastric vaccines [40]. Interestingly, one out of six mice from the IN:Spore group showed anti-PlpE IgA and anti-NLKT IgA in BAL samples, though not in other samples. While difficult to explain, it is noteworthy that this outcome is not unique, as other studies have reported similar findings where antigen-specific antibody production has been reported from spore-only immunization [41,42]. Studies have also reported that spores alone can provide protection against H5N1 virus challenge [19] and lethal *B. anthracis* challenge [42] in mouse models. The reported underlying mechanism was the involvement of innate immunity via TLR-mediated NF-ĸB expression, the recruitment of NK cells to the lungs, and dendritic cell maturation [19,43].

Similar to Spore-MhCP1, immunization with Spore-MhCP2 stimulated detectable anti-LKT and anti-GS60 antibodies, demonstrating that the chimeric antigen MhCP2 also retained immunogenicity after spore adsorption. While the intramuscular immunization of MhCP2 demonstrated a stronger serum IgG immune response compared to other treatment groups, the intranasal immunization of spore-bound and free MhCP2 also induced seroconversion against both antigen constructs, LKT and GS60, independently. Between these two intranasal treatment groups, the spore-bound MhCP2 more efficiently elicited a systemic immune response, yielding a higher IgG antibody titre. GS60 has previously been targeted for vaccine development in the form of transgenic alfalfa expressing GS60 in rabbits [28] and recombinant GS60 in calves [44] and was reported to be immunogenic. Although GS60 is expressed in vivo during bronchopneumonia disease progression and is conserved among several BRD pathogens [28], there are very few reported vaccination trials testing this antigen [28,44]. Orouji et al. (2012) reported that the elevated IgG antibody titre against GS60 correlated with a reduction in the percentage of pneumonic tissue, a potential association between protection and anti-GS60 IgG [44]. However, none of these studies reported any antibody levels at mucosal sites.

The intranasal Spore-MhCP2 immunization of mice displayed the strongest anti-LKT and anti-GS60 IgA in all mucosal samples (BAL, feces, and saliva) evaluated. The intranasal immunization of free MhCP2 also generated IgA antibodies in all samples, albeit at lower levels than the spore-bound MhCP2. This indicates that binding MhCP2 to *Bacillus* spores did result in an increased immune response, again highlighting that the spores were effective as an adjuvant. Also similar to Experiment 1, it was shown that intranasal immunization resulted in mucosal immunity in the oral cavity and gastrointestinal tract. None of the spore-only treatment mice in Experiment 2 exhibited detectable antibody responses, in contrast to Experiment 1. The intragastric administration of spore-bound MhCP2 induced limited levels of anti-LKT IgA in BAL, saliva, and fecal samples. This was in contrast to Experiment 1, where IG:Spore+MhCP1 did not elicit detectable mucosal immunity in BAL and oral samples. It was also apparent that MhCP2 in Experiment 2 resulted in greater antigen-specific IgA at all mucosal sites. While this may have been partly due to the antigen itself, the differences in the amount of antigen administered between the two experiments (10 µg of MhCP1 versus 50 µg of MhCP2) likely also contributed to the observed differences. Recently, our group has developed a plant-based oral vaccine and reported the induction of both systemic and mucosal immune responses in mice after five vaccinations [45]. It is important to consider the differences in vaccine delivery methods, antigen concentration, and the number of doses administered when evaluating vaccines, as these factors can impact immunity outcome. Since intranasally delivered Spore-MhCP2 elicited both mucosal and systemic immune responses in mice, it could provide substantial protection against *M. haemolytica* infection in cattle, particularly given the stronger response observed compared to Spore-MhCP1.

Complement-mediated serum bactericidal activity assay is considered a standard method for evaluating the capability of antibodies induced by a vaccine to kill the targeted pathogen [46]. In both mouse experiments, vaccine-induced antibodies from the intramuscular treatment demonstrated an almost complete killing of *M. haemolytica* in the complement-dependent SBA assay. This outcome aligns with previous findings where bactericidal activity was noted following the intramuscular vaccination of mice using a comparable construct to MhCP1 [30,34]. Although the sera from intranasal Spore-MhCP1 and free MhCP1 treatment mice exhibited a lower level of bactericidal activity in comparison to intramuscular treated mice in Experiment 1, this was likely attributed to lower IgG levels following intranasal immunization [47]. In Experiment 2, this was also evident as we observed an elevated level of bactericidal activity from these two intranasal treatment mice groups which also demonstrated a better IgG titre compared to Experiment 1. Surprisingly, although intragastric administration failed to demonstrate any detectable range of serum IgG for spore-bound MhCP1 and limited IgG for spore-bound MhCP2, sera from these groups had bactericidal activity. This finding features an important observation that the spore-bound MhCP1 and MhCP2 antigens had the capability to elicit functional antibodies against *M. haemolytica* whether it was delivered intranasally or intragastrically. Even though oral administration failed to elicit appreciable immune responses in mice, it is justifiable to explore the assessment of oral spore-based vaccines in ruminants due to anatomical distinctions between mice and cattle and the prospect of rumination, which could lead to the activation of pharyngeal lymphoid tissue by the exposure of antigens via regurgitation [10]. If similar IgA and IgG production occurs in cattle after vaccination with Spore-MhCP1/MhCP2, this would be advantageous by limiting the colonization and proliferation of *M. haemolytica* in the upper respiratory tract and potentially reducing infection in the lungs, respectively [3,10].

## 5. Conclusions

In summary, the intranasal immunization of mice with both Spore-MhCP1 and Spore-MhCP2 resulted in mucosal and systemic antibody production. These responses were notably heightened in comparison to the intranasally delivered free antigen, demonstrating that binding antigens to *Bacillus* spores did result in an increased immune response. This observation emphasizes the effectiveness of spores as an adjuvant in augmenting the immune response and highlights their potential use in the development of novel immunization strategies against infectious diseases. It is interesting to note that the intragastric delivery of spore-bound vaccines conferred partial systemic protection, as evidenced by SBA, despite limited IgG responses. The outcomes from the mouse experiments in this study suggest that the employment of a similar approach could provide substantial protection against *M. haemolytica* infection in cattle and warrant further investigations to assess the effectiveness of the *B. subtilis* spore-based mucosal vaccines in cattle.

## Figures and Tables

**Table 1:** Design of mice experiments (Exp) in which BALB/c mice were immunized with *Mannheimia haemolytica* chimeric protein MhCP1 (CTB+PlpE+NLKT; Exp. 1) or MhCP2 (CTB+LKT+GS60; Exp. 2) in free form or bound to *B. subtilis* spores.

Exp. No.	Treatment Group	No. of Mice	Route of Administration	Vaccine Formulations (Amount per Dose)	Vaccination Days	Samples Collected
Exp. 1	IM:MhCP1	12	Intramuscular	MhCP1 (10 µg) mixed with IFA *; No Spore	Days 0 and 21	Blood, BAL ^#^, feces, saliva
	IN:Spore+MhCP1	12	Intranasal	Spore (2 × 10^9^)-bound MhCP1 (10 µg)	Days 0 and 21	Blood, BAL, feces, saliva
	IN:MhCP1	12	Intranasal	MhCP1 (10 µg) only; No Spore	Days 0 and 21	Blood, BAL, feces, saliva
	IN:Spore	12	Intranasal	Spore (2 × 10^9^) only; No MhCP1	Days 0 and 21	Blood, BAL, feces, saliva
	IG:Spore+MhCP1	12	Intragastric	Spore (2 × 10^9^)-bound MhCP1 (10 µg)	Days 0 and 21	Blood, BAL, feces, saliva
	Control	12	N/A	No MhCP1; No Spore	Days 0 and 21	Blood, BAL, feces, saliva
Exp. 2	IM:MhCP2	12	Intramuscular	MhCP2 (50 µg) mixed with IFA; No Spore	Days 0 and 21	Blood, BAL, feces, saliva
	IN:Spore+MhCP2	12	Intranasal	Spore (2 × 10^9^)-bound MhCP2 (50 µg)	Days 0 and 21	Blood, BAL, feces, saliva
	IN:MhCP2	12	Intranasal	MhCP2 (50 µg) only; No Spore	Days 0 and 21	Blood, BAL, feces, saliva
	IN:Spore	12	Intranasal	Spore (2 × 10^9^) only; No MhCP2	Days 0 and 21	Blood, BAL, feces, saliva
	IG:Spore+MhCP2	12	Intragastric	Spore (2 × 10^9^)-bound MhCP2 (50 µg)	Days 0 and 21	Blood, BAL, feces, saliva
	Control	12	N/A	No MhCP2; No Spore	Days 0 and 21	Blood, BAL, feces, saliva

* IFA: Incomplete Freund′s adjuvant, ^#^ BAL: bronchoalveolar lavage.

## Data Availability

The datasets used in the current study are available from the corresponding author upon reasonable request.

## Supplementary Materials

The following supporting information can be downloaded at https://www.mdpi.com/article/10.3390/vaccines12040375/s1, Figure S1: Adsorption of *Mannheimia haemolytica* chimeric proteins MhCP1 and MhCP2 to *Bacillus subtilis* spores using optimized binding conditions; Figure S2: Experiment 1—Antigen-specific antibody responses measured by enzyme-linked immunosorbent assay (ELISA) from samples collected on day 21; Figure S3: Experiment 2—Antigen-specific antibody responses measured by enzyme-linked immunosorbent assay (ELISA) from samples collected on day 21.
