# Supplementary material for: Mucosal Immunization with Spore-Based Vaccines against Mannheimia haemolytica Enhances Antigen-Specific Immunity
Source: Vaccines (Basel). 2024 Apr 1;12(4):375. doi: 10.3390/vaccines12040375 (PMC11054499; doi:10.3390/vaccines12040375)
Supplement: Supplementary file 1 [file vaccines-12-00375-s001.zip › vaccines-2889407-supplementary.pdf]

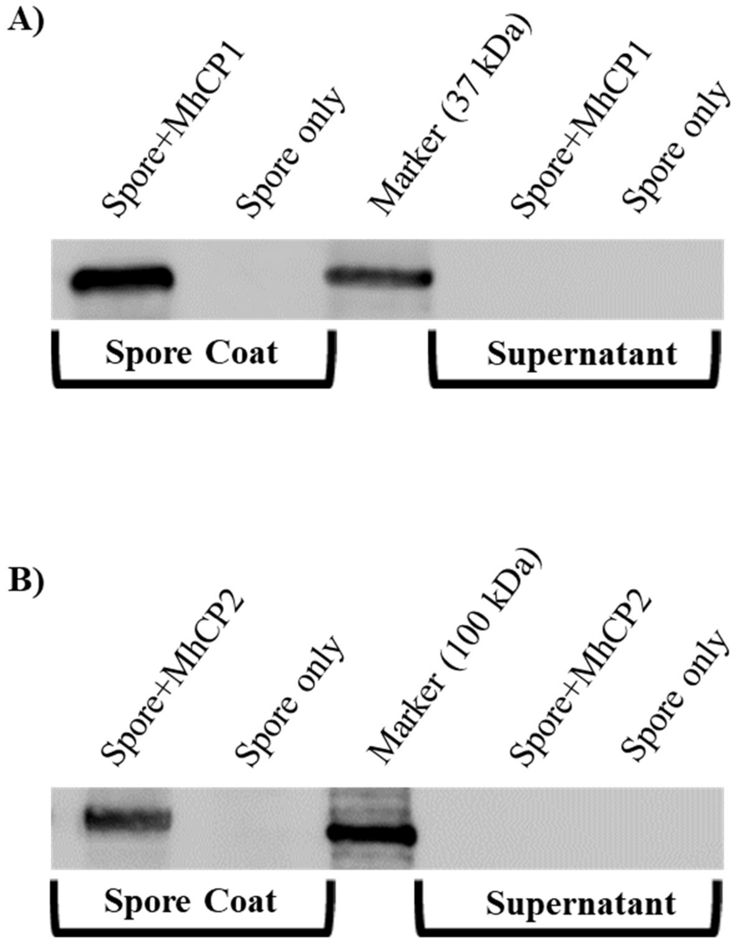

Supplementary Figure S1. The adsorption of *Mannheimia haemolytica* chimeric proteins MhCP1 and MhCP2 to *Bacillus subtilis* spores using optimized binding conditions. A) The optimized adsorption of MhCP1 to *Bacillus subtilis* spores demonstrated by Western blotting. Briefly, 10 µg of MhCP1 was mixed with 2 x 10^9 spores in phosphate buffered saline (PBS) at pH 4 and incubated for 1 h at 4 °C. The binding mixture was centrifuged, the supernatant collected, and the pellet was washed two times with PBS. Pellets were resuspended in 100 µl of spore coat extraction buffer, then incubated at 65 °C for 30 min to remove spore coat proteins from the spores. Using a one in ten dilution of the extraction, detection was confirmed by the Western blotting of size-fractionated proteins. B) The optimized adsorption of MhCP2 to *B. subtilis* spores demonstrated by Western blotting. Briefly, 50 µg of MhCP2 was mixed with 2 x 10^9 spores in citrate buffer at pH 4 and incubated for 1 h at 4 °C, then the same procedures described above were followed.

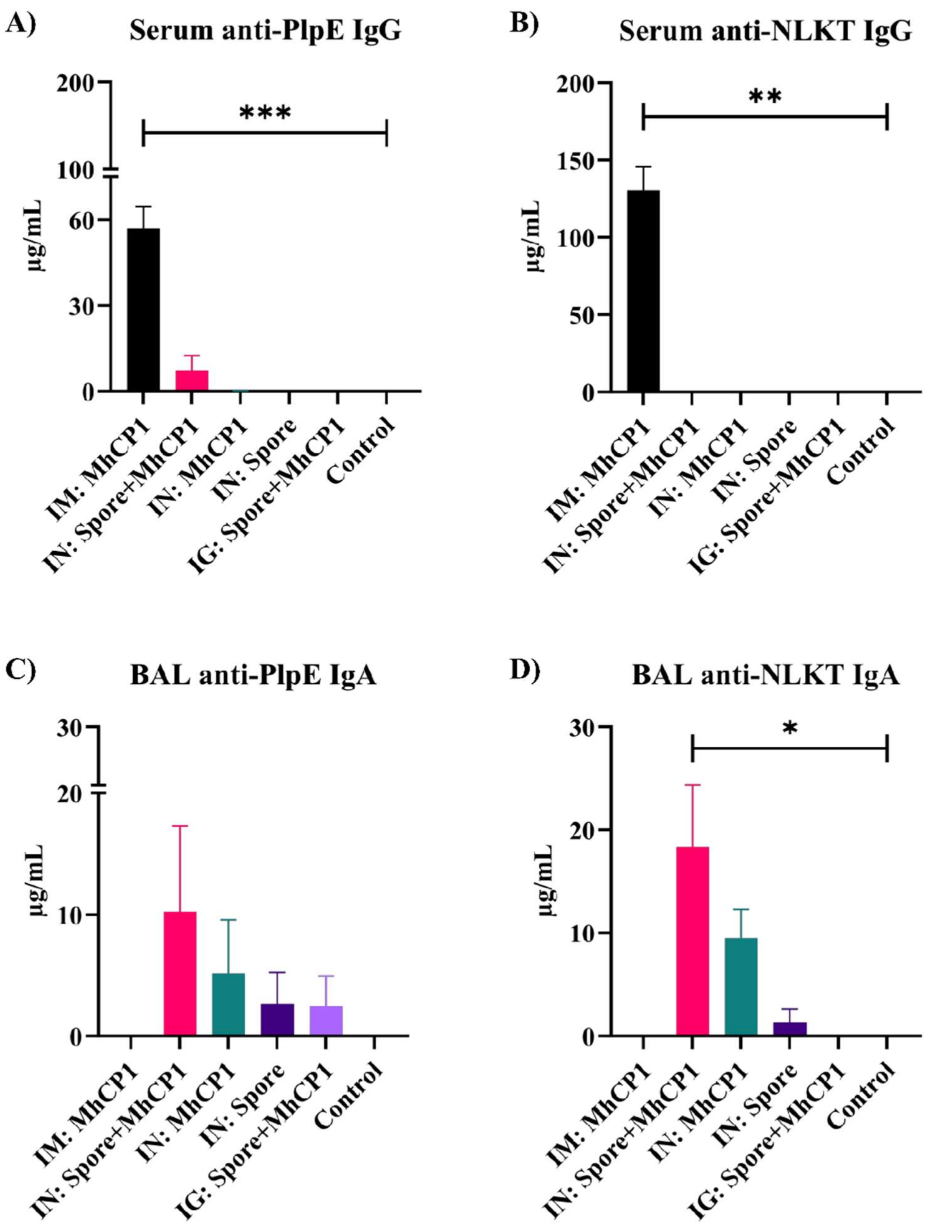

Supplementary Figure S2. Experiment 1—Antigen-specific antibody responses measured by enzyme-linked immunosorbent assay (ELISA) from samples collected on day 21. ELISA plates coated with either recombinant PlpE or NLKT were used to measure anti-PlpE and anti-NLKT antibodies in mice sera and bronchoalveolar lavage (BAL). Immune responses from six experimental groups: intramuscular (IM:MhCP1), intranasal spore-bound antigen (IN:Spore+MhCP1), intranasal antigen only (IN:MhCP1), intranasal spore only (IN:Spore), oral/intragastric spore-bound antigen (IG:Spore+MhCP1), and control/naïve mice (Control) were compared. (A) Levels of serum IgG specific to PlpE. (B) Levels of serum IgG specific to NLKT. (C) Levels of secretory IgA specific to PlpE from BAL. (D) Levels of secretory IgA specific to NLKT from BAL. Results are expressed as mean ± SEM. Significance was tested against the control by one-way ANOVA with Tukey's multiple comparison test, *p < 0.05, ** p < 0.01, and ***p < 0.001.

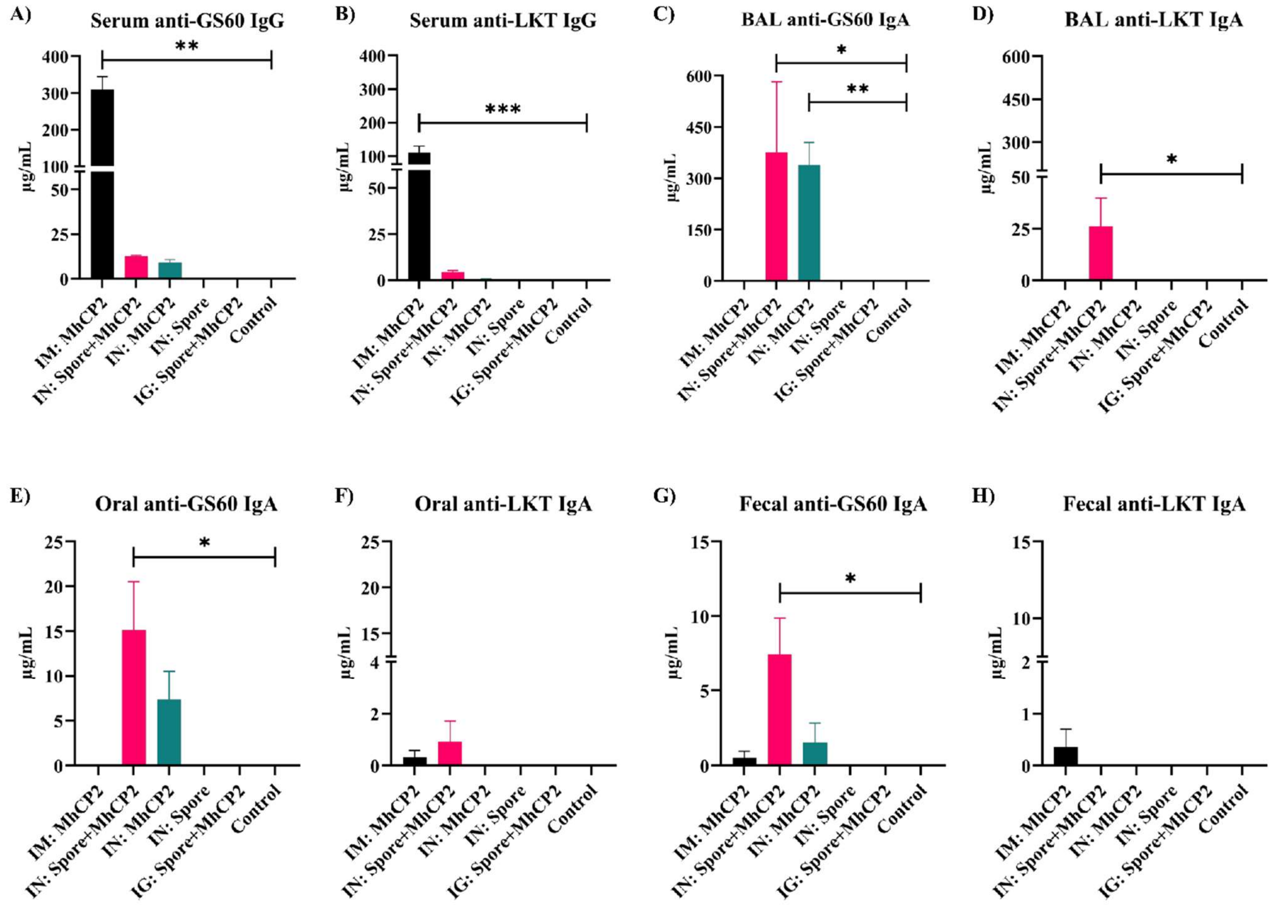

Supplementary Figure S3. Experiment 2—Antigen-specific antibody responses measured by enzyme-linked immunosorbent assay (ELISA) from samples collected on day 21. ELISA plates coated with either recombinant GS60 or LKT were used to measure anti-GS60 and anti-LKT antibodies in mice sera, bronchoalveolar lavage (BAL), saliva, and fecal samples. Immune responses from six experimental groups: intramuscular (IM:MhCP2), intranasal spore-bound antigen (IN:Spore+MhCP2), intranasal antigen only (IN:MhCP2), intranasal spore only (IN:Spore), oral/intragastric spore-bound antigen (IG:Spore+MhCP2), and control/naïve mice (Control) were compared. (A) Levels of serum IgG specific to GS60. (B) Levels of serum IgG specific to LKT. (C) Levels of secretory IgA specific to GS60 from BAL. (D) Levels of secretory IgA specific to LKT from BAL. (E) Levels of secretory IgA specific to GS60 from saliva. (F) Levels of secretory IgA specific to LKT from saliva. (G) Levels of secretory IgA specific to GS60 from feces. (H) Levels of secretory IgA specific to LKT from feces. Results are expressed as mean ± SEM. Significance was tested against the control by one-way ANOVA with Tukey's multiple comparison test, *p < 0.05, ** p < 0.01, and ***p < 0.001.
